# How do brassinosteroids fit in bud outgrowth models?

**DOI:** 10.1093/jxb/erad394

**Published:** 2023-10-17

**Authors:** Jack H Kelly, Philip B Brewer

**Affiliations:** Waite Research Institute, School of Agriculture Food & Wine, The University of Adelaide, Adelaide, SA 5064, Australia; Waite Research Institute, School of Agriculture Food & Wine, The University of Adelaide, Adelaide, SA 5064, Australia; Australian Research Council Training Centre for Future Crops Development, The University of Adelaide, Adelaide, SA 5064, Australia; Australian Research Council Centre of Excellence for Plant Success in Nature and Agriculture, The University of Queensland, Brisbane, QLD 4072, Australia; University of Sydney, Australia

**Keywords:** Auxin, brassinosteroid, bud outgrowth, crop architecture, cytokinin, gibberellin, strigolactone, *TB1*

## Abstract

A network of plant hormonal signals coordinates plant branching. Brassinosteroids are important in this network, acting as repressors of the strigolactone pathway and *TEOSINTE BRANCHED1* .

**Short stature crops were developed during the green revolution mainly due to their resistance to falling over (lodging), improved crop harvestability and management, and a greater proportion of biomass in the grains, leading to superior yield. These crops were disrupted in the gibberellin (GA) pathway, which caused the reduced height (** **Gao and Chu, 2020** **). GA disruption can introduce unwanted effects in other important traits such as fertility, leaf expansion, seed quality, and stress response (** **Gao and Chu, 2020** **). Hence, there are currently efforts to uncouple negative side effects of GA-related short stature or utilize alternative dwarfing pathways, such as brassinosteroids (BRs).**

A negative side effect of GA-related short stature in grain crops can be increased tillering, leading to unproductive tillers and small grains (Kertesz *et al.*, 1991). This is probably because the GA pathway affects a range of traits that help the plant to adapt to its environment, which can have positive or negative impacts on plant growth and yield. Short stature with unchanged tillering would be preferable. To achieve optimal height and tiller number, it will be essential to have a full understanding of how hormonal, resource, and environmental signals act on stature and bud outgrowth. Although models for bud outgrowth regulation have been proposed (Beveridge *et al.*, 2023), they still lack some important information, such as why increased tillering occurs in GA-deficient plants.

BRs have emerged as a plant hormone that also influences stature and bud outgrowth (Xia *et al.*, 2021), and they have become a key piece of the bud outgrowth puzzle. For instance, the rice BR mutant *dwarf and low tillering* (*dlt*) exhibits reduced stature coupled with reduced tillering (Tong *et al.*, 2009). Understanding how BRs integrate within the branching signalling network may be important for decoupling tillering from height and achieving optimal crop shoot architecture.

## *TB1* integrates multiple signals to regulate bud outgrowth

The decision in determining whether a bud is released or held in a state of dormancy is regulated by a complex network of hormonal signals (Kelly *et al.*, 2023). Many of these signals act via interactions with *TEOSINTE BRANCHED1* (*TB1*), a transcription factor gene that inhibits bud outgrowth in monocotyledons [known as *BRANCHED1* (*BRC1*) in dicotyledons]. An overview of the key interactors is shown in Box 1, but more details can be found in recent reviews (Beveridge *et al.*, 2023; Dun *et al.*, 2023; Kelly *et al.*, 2023). Strigolactones (SLs) are a key inhibitor of branching, whereby promoting the expression of *TB1* then can repress axillary bud outgrowth (Dun *et al.*, 2012). Cytokinins (CKs) are also important, acting antagonistically to SLs by repressing SL biosynthesis and *TB1* expression in buds to promote branching (Dun *et al.*, 2012). While GA has been selected for modifying plant height, findings in *Rosa* sp. show that GA biosynthesis in buds becomes up-regulated during bud burst, suggesting that GA also plays a positive role in branching (Choubane *et al.*, 2012). This aligns with findings that highlight a direct repressive effect of GA on *TB1* (Ni *et al.*, 2015). However, GA also represses SL biosynthesis, which might be expected to promote *TB1* and subsequently reduce branching (Ito *et al.*, 2017). However, findings in tomato show a repressive effect of GA on CK response (Fleishon *et al.*, 2011). Hence, it may be that targeting dampened GA for reduced height can result in increased CK levels, which may contribute to increased tillering in GA-related semi-dwarf crops. Moreover, soluble sugars may over-ride the effect of low GA on SL biosynthesis, as sucrose also promotes branching through the DWARF53 (D53) suppressor of SL signalling (Dun *et al.*, 2023). In Arabidopsis, abscisic acid (ABA) gene expression is up-regulated in wild-type plants treated with far-red light, while *brc1* mutants exhibit no response (Gonzalez-Grandio *et al.*, 2013). This suggests that ABA acts downstream of *TB1*, where it is promoted to repress bud outgrowth and maintain bud dormancy (Box 1).

Box 1. **BRs integrate *TB1* to regulate bud outgrowth**

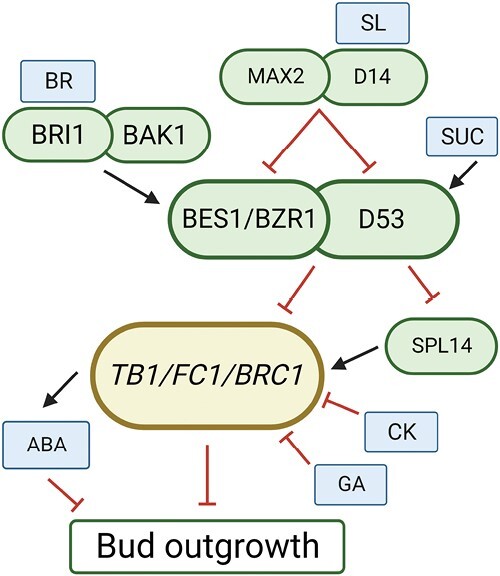

BR perception via receptor BRASSINOSTEROID INSENSITIVE 1 (BRI1) and co-receptor BRI1-ASSOCIATED RECEPTOR KINASE 1 (BAK1) promotes increased BES1/BZR1 levels. BZR1 interacts with D53 to repress *TB1*, which promotes bud outgrowth. MAX2-mediated BES1 degradation is promoted by SLs, which de-repress *TB1* and inhibit bud outgrowth. D53 also represses *SQUAMOSA PROMOTER BINDING PROTEIN LIKE 14* (*SPL14*), a positive regulator of *TB1*. BR, CK, SUC, and GA promote branching, while SL represses branching by promoting *TB1.* ABA is promoted by *TB1*, acting downstream to negatively regulate branching. Created with BioRender.com.

By promoting SL biosynthesis and repressing CK biosynthesis, auxin can modulate *TB1* levels to regulate branching responses (Beveridge *et al.*, 2023). This is part of the mechanism of apical dominance, whereby auxin synthesized in a growing apex (shoot tip) maintains dormancy in nearby buds and allows buds to grow out if the shoot tip is removed (Beveridge *et al.*, 2023). Auxin also promotes the expression of *PIN-FORMED* (*PIN*) auxin transporters and their localization at the plasma membrane towards cells in the stem where auxin is depleted (Beveridge *et al.*, 2023). This allows auxin to flow from actively growing leaves (sources) to established channels of auxin flow in the stem vasculature (sinks). This flow triggers new vasculature formation needed to support a growing shoot. Auxin flowing within the stem can also prevent auxin canalization from an axillary bud, potentially inhibiting ongoing outgrowth independently of *TB1* (Waldie and Leyser, 2018). Elevated PIN1 levels in SL biosynthesis mutants also suggest that SLs act to inhibit auxin transport (Waldie and Leyser, 2018). This decreases the sink strength of auxin flow in the main stem, thus inhibiting new auxin canalization from lateral sources, such as buds (Waldie and Leyser, 2018; Zhang *et al.*, 2020). Moreover, CK can modulate PIN levels, suggesting that it may also act on auxin canalization from buds (Waldie and Leyser, 2018).

These findings highlight *TB1* as a hub for shoot branching together with *TB1*-independent pathways. Understanding the details of the model may provide ideas for changing plant architecture, such as optimizing tillering in reduced-stature crops. However, it is essential to also include other important factors, as these may be needed to solve conflicts in the model.

## How do brassinosteroids function in this network?

When observing BR mutant phenotypes, it is clear that BRs play an important role in regulating branching and stem elongation. The question is: how do they carry out this effect? It has been previously reported in Arabidopsis that the BR signalling component BRI-EMS SUPPRESSOR-1 (BES1) interacts with the key SL signalling component MORE AXILLARY GROWTH 2 (MAX2), which results in the degradation of BES1 (Wang *et al.*, 2013). It has also been observed that SL-mediated tiller responses in rice are dependent on levels of the BES1 homologue BRASSINAZOLE-RESISTANT 1 (BZR1) (Fang *et al.*, 2020). SL and BR double mutants produce fewer tillers than the *dwarf14* (*d14*) SL-insensitive single mutant, suggesting that upstream BR signalling or downstream BZR1 function is required for D14-regulated tillering response (Fang *et al.*, 2020). BZR1 also interacts with D53 to coordinate both SL- and BR-mediated tiller responses in rice, where BZR1 recruits D53 to the promoter of the *TB1* rice homolog *FINE CULM1* (*FC1*) to regulate its expression (Fang *et al.*, 2020).

Xia *et al.* (2021) further explored the relationship between BRs and *TB1*/*BRC1*, observing that decapitation in tomato resulted in increased levels of BL and BZR1; however, *BRC1* was not as significantly repressed in *dwf* and *bzr1* mutants. This suggests that BRs are required for *BRC1* repression to release bud outgrowth. Additionally, mutation of *brc1* or overexpression (OE) of *DWF* in tomato both enhanced bud outgrowth; however, *brc1* in *DWF-OE* plants did not further increase bud outgrowth, suggesting that *BRC1* acts downstream to regulate BR-mediated branching responses (Xia *et al.*, 2021). Xia *et al.* also conducted multiple assays in tomato to examine if BZR1 directly regulates *BRC1* expression, where they observed multiple potential binding sites for BZR1 in the *P*_*BRC1*_-bait vector, and that *P_BRC1_* was inhibited by BZR1 binding (Xia *et al.*, 2021). This suggests that BR signalling positively regulates bud outgrowth by down-regulating *BRC1* expression via BZR1 (Box 1). Thus, BRs and SLs appear to act antagonistically on *TB1*/*BRC1* expression by, respectively, promoting or inhibiting BES1/BZR1. Importantly, these key findings cement BRs firmly into the branching network. It has also been recently observed that light-mediated effects on bud outgrowth are partly BR dependent. Dong *et al.* (2023) observed lower transcript levels of *DET2* and *DWF* and reduced levels of BL and BZR1 in lateral buds in response to red/far-red (R/FR) light, causing up-regulation of *BRC1* and repression of bud outgrowth in tomato. This response is regulated by LONG HYPOCOTYL 5 (HY5), which can bind the promoters of *BRC1*, *DET2*, and *DWF* to dampen *BRC1* levels and up-regulate BR biosynthesis to enhance shoot branching (Dong *et al.*, 2023).

## Outlook

While we highlight results showing significant direct effects of BRs on the SL and *TB1* pathway, what remains less understood are the effects of BRs on the other branching hormones. This will perhaps help solve some discrepancies in the model and increase our ability to refine or uncouple genetic outputs for crop architecture improvement. Modifications to crop shoot architecture have played a significant role in boosting productivity, which has largely been facilitated by targeting hormone pathways such as GA. As hormones regulate many aspects of plant function and development, stronger mutations of hormonal function can introduce unwanted effects, such as elevated tillering associated with dampened GA. Hence it is important to continue expanding our understanding of the fundamental mechanisms that regulate height and tillering, as this may elucidate new alternative targets for modifying shoot architecture. BRs promote stem elongation and tillering, in part by dampening *TB1*. Therefore, biosynthesis or signalling components may serve as ideal targets for genetic manipulation. This can be possible using a quantitative trait engineering approach, where candidate genes can be targeted to uncover intermediate (hypomorphic) alleles that promote beneficial crop architecture. These findings highlight that the BRs are another core component of the complex network that regulates tillering.
